# Manchette-acrosome disorders during spermiogenesis and low efficiency of seminiferous tubules in hypercholesterolemic rabbit model

**DOI:** 10.1371/journal.pone.0172994

**Published:** 2017-02-27

**Authors:** Layla Simón, Abi K. Funes, Martín A. Yapur, María E. Cabrillana, María A. Monclus, Paola V. Boarelli, Amanda E. Vincenti, Tania E. Saez Lancellotti, Miguel W. Fornés

**Affiliations:** 1 Laboratorio de Investigaciones Andrológicas de Mendoza (LIAM). Instituto y Área de Histología y Embriología (IHEM), Facultad de Ciencias Médicas, Universidad Nacional de Cuyo y Centro Científico Tecnológico (CCT), Consejo Nacional de Investigaciones Científicas y Técnicas (CONICET), Mendoza, Argentina; 2 Instituto de investigaciones. Facultad de Ciencias Médicas. Universidad del Aconcagua. Mendoza, Argentina; Universite Blaise Pascal, FRANCE

## Abstract

Hypercholesterolemia is a marker for several adult chronic diseases. Recently we demonstrated that sub/infertility is also associated to Hypercholesterolemia in rabbits. Seminal alterations included: abnormal sperm morphology, decreased sperm number and declined percentage of motile sperm, among others. In this work, our objective was to evaluate the effects of hypercholesterolemia on testicular efficiency and spermiogenesis, as the latter are directly related to sperm number and morphology respectively. Tubular efficiency was determined by comparing total number of spermatogenic cells with each cell type within the proliferation/differentiation compartments. We found lower testicular efficiency related to both a decrease in spermatogonial cells and an increase in germ cell apoptosis in hypercholesterolemic rabbits. On the other hand, spermiogenesis–the last step of spermatogenesis involved in sperm shaping–was detaily analyzed, particularly the acrosome-nucleus-manchette complex. The manchette is a microtubular-based temporary structure responsible in sperm cell elongation. We analyzed the contribution of actin filaments and raft microdomains in the arrangement of the manchette. Under fluorescence microscopy, spermatocyte to sperm cell development was followed in cells isolated from V to VIII tubular stages. In cells from hypercholesterolemic rabbits, abnormal development of acrosome, nucleus and inaccurate tail implantation were associated with actin–alpha-tubulin–GM1 sphingolipid altered distribution. Morphological alterations were also observed at electron microscopy. We demonstrated for the first time that GM1-enriched microdomains together with actin filaments and microtubules are involved in allowing the correct anchoring of the manchette complex. In conclusion, cholesterol enriched diets promote male fertility alterations by affecting critical steps in sperm development: spermatogenesis and spermiogenesis. It was also demonstrated that hypercholesterolemic rabbit model is a useful tool to study serum cholesterol increment linked to sub/infertility.

## Introduction

Hypercholesterolemia (HC) triggers deleterious effects on several tissues defining an important medical and epidemiologic entity [1]. Recently, it has also been reported together with obesity as a risk factor for male infertility [2, 3]. Although several studies have evaluated the effect of HC on semen quality, there are few reports concerning the influence of cholesterol-enriched diets on spermatogenesis. We developed a HC animal model to assess the influence of cholesterol-enriched diets on sperm defects occurring during spermatogenesis.

We previously reported that the administration of a fat diet to healthy rabbits promotes seminal alterations: decrease in semen volume and increase in sperm morphological abnormalities, among others [4]. Other authors also demonstrated that serum lipids affect semen quality parameters, specifically sperm head morphology [5]. But the underneath mechanism remain elusive.

Seminal volume decrease was associated with a decline in total sperm count. The reduced number of sperm could be explained by low testicular efficiency (sperm decreased production by seminiferous tubule) [6, 7]. This parameter could be studied tabulating the spermatogenic cells related to the tubular compartments (proliferation and differentiation) and seminal sperm number [8]. On the other hand, changes in sperm morphology could be related to seminiferous tubules dysfunction, due to abnormal spermiogenesis [9]. Sperm head development is achieved by the action of an acroplaxome-manchette complex during nuclear remodeling and acrosome assembly [10, 11]. Any interference with this intracellular machinery could result in abnormal head shaping [11].

Lipid rafts are cholesterol and sphingolipid-enriched domains of cell membranes [12]. Interactions between this highly “lipid-ordered” microdomains and cytoskeletal components can contribute to the regulation of raft assembly/clustering and cytoskeletal dynamics [13, 14]. It is fairly known that cholesterol depletion disrupts raft structure [15]. However, recent studies suggest that cholesterol enrichment could also alter raft structure leading to abnormal cell function [16, 17]. At present the link between lipid rafts and the anchoring of acroplaxome-manchette complex to plasma membrane is unknown.

In this paper we investigated the causes of fat-induced seminal alterations: reduced sperm number and abnormal morphology in adult rabbits

## Materials and methods

### Ethics statement

Animal studies described here were reviewed and approved by the animal care and use committee of School of Medicine, National University of Cuyo (Institutional Committee for Use of Laboratory Animals, IACUC- http://fcm.uncuyo.edu.ar/paginas/index/cicual); protocol reference number: 06_150702.

### Reagents

Unless otherwise stated, all chemicals and solvents of the highest grade available were obtained from Sigma (St. Louis, MO, USA) and Merck (Darmstadt, Germany).

Euthanyle®: Product authorized by SENASA—http://www.senasa.gov.ar, government animal health regulation. Formula: 40 g pentobarbital /100ml and 5 g diphenylhydantoin /100 ml (Brouwer laboratory S.A., Argentina).

Phosphate buffer saline (PBS): was prepared following the manufacturer instructions, dissolving one tablet in 100 ml of double distilled water, preparing 1X PBS solution containing 137 mM sodium chloride, 2.7 mM potassium chloride, and 10 mM phosphate buffer, pH: 7.3–7.5 (MP Biomedicals, California, USA).

First bovine juice: ‘‘Primer jugo bovino”, commercial preparation (composed by 55% saturated fat, Juan Lopez y CIA.), Argentina Alimentary Code (http://www.anmat.gov.ar/alimentos/codigoa/CAPITULO_VII.pdf; article 543 –resolution 2012, 19.10.84).

### Animals and diets

Ten fertile male rabbits (White New Zealand, 6 months old) were acquired from rabbit farm (Verde-Azul farm, Buenos Aires—Argentina). During 12 months, animals were caged and maintained with a photoperiod of 12 hours light / day and a temperature ranging from 18–25°C. Rabbits were divided in two groups of 5 animals each. The first group (designated normal cholesterolemic rabbits, NCR) was fed *ad libitum* with a standard rabbit diet (GEPSA FEEDS, Buenos Aires–Argentina: 17% crude protein, 16% fiber, 2% minimal ether extract: 0% saturated fat, 5.3% minerals). The second group (hypercholesterolemic rabbits) was fed *ad libitum* with fat-enriched diet following our previous animal model [4]. This group was named HCARDA: Hypercholesterolemia acquired by acutely feeding adult rabbits with standard diet supplemented with cow grease. This experimental diet (ED) was prepared by heating (up to 60°C) 200 g of fat derived from cow; primer jugo bovino. Melted oil was poured over 1.5 kg of stock diet and mechanically mixed. The resulting stock ED was enriched up to 0.05% cholesterol (cholesterol enrichment was determined by chromatography analysis at National Institute of Industrial Technology, INTI—Argentina).

### Tissue collection

Rabbits were sacrificed by a lethal dose of pentobarbital (1 ml/5 kg; Euthanyle) via pinna marginal veins. After sacrifice, entire testicles were collected in PBS. Some samples were separated, cut in small cubes and fixed for light or transmission electron microscopy. The remaining tissue was decapsulated with scissors and the seminiferous tubules were transferred to a fresh PBS buffer containing collagenase (see below).

### Serum cholesterol

After 3 months of fat diet, blood was obtained fortnightly from marginal ear vein with heparinized 1 ml syringes. Immediately after bleeding, blood was centrifuged at 1,100 *g* by 10 minutes in a clinical centrifuge. Supernatant was carefully aspirated and aliquots were processed using GTlab kit (GTlab, Rosario, Argentina) to determine cholesterol levels.

### Semen assays

Ejaculated semen was collected by an artificial vagina from both NCR and HCARDA twice a month [18]. Semen samples were stored at 37°C, assayed for volume, aspect, color, pH, sperm motility and cell concentration, as previously stated [4]. Total count of spermatozoa per ejaculate was calculated with semen from the last two months of experimental time. Remaining semen sample was washed in PBS twice by centrifuging at 750 *g* by 10 min and the final pellet was resuspended in fixative solution (4% paraformaldehyde in PBS). Then, smears of fixed sperms were stained by Giemsa and morphological abnormalities were tabulated [19]. Morphological abnormalities were classified according to the type of sperm head alteration or the relation between head and tail sperm axis.

### Light microscopy

Following standard procedures, histological analyses were performed on testicular tissue sections. Briefly, small pieces of testis obtained from NCR and HCARDA were fixed with formalin (10% formol), dehydrated in ethanol-xylene and embedded in paraffin. Sections of 5 μm thicknesses were obtained on a sliding microtome, mounted on slides, dewaxed with xylene and stained with hematoxilin-eosin. Slides were examined by light microscopy (Nikon 80i). Identification and characterization of spermatogenic cells—from spermatogonium to elongated spermatids–was achieved following cell morphology patterns. Then, the percentage of different cell types was calculated (see testicular efficiency measurement).

### Transmission electron microscopy

Small pieces of testis from both experimental conditions were fixed with 4% para-formaldehyde (w/v), 4% glutaraldehyde and 20% picric acid (v/v) saturated in PBS [20] and post-fixed with 1% OsO4 (w/v) overnight at 4°C. Osmified tissues were dehydrated in ethanol-acetone (up to absolute acetone) and embedded in epoxy resin (Epon 812, Pelco—EEUU). Ultra-thin sections were obtained by Ultracut equipment (Leitz), stained with classical uranyl acetate and lead citrate and examined with a Zeiss EM 900 microscopy (Zeiss, Oberkochen, Germany).

### Spermatogenic cells isolation

Testes were collected and the tunica albuginea was removed with sterile forceps and discarded. The seminiferous tubules were treated with collagenase for 10 minutes (5 mg/ml of collagenase from *Clostridium histolyticum*, type IV, SIGMA C5138). Cycles of the seminiferous epithelium described by Swierstra [21] were detected using the trans-illumination method [22]. Stages V to VIII were selected, based on Golgi-acrosome complex development. Seminiferous tubules were cut under estereomicroscope observation and placed on slides at room temperature. Seminiferous cells were extruded out by both tubule ends applying slight pressure with a coverslip [23]. Then, after checking cells presence under a microscope (40x), 40 μl of fixative solution were applied onto slides for 20 minutes. Fixed cells were reserved protected from light and dust for future treatments.

### Acrosomal asymmetry measurement: Asymmetry index

Spermatogenic cells were stained with Toluidine Blue for 60 seconds and observed by light microscopy. The symmetry in acrosome development was studied by measuring the distance from the central axis to each acrosomal end (S1 Fig), compared to the total distance between both ends. Central axis is an imaginary line passing through the acrosomal granule and implantation fossa (tail-head union).

$$Asymmetry\;index=\frac{1-2}{1+2}\times 100$$

When 1 and 2 are equal, the index is 0. The measurements were performed by Image J free software (http://imagej.nih.gov/ij/).

### Oil red O lipid droplets staining

Fixed spermatogenic cells were washed with PBS and incubated 5 minutes with isopropanol 60% in order to detect lipid storages. Staining was performed by covering cells 5 minutes with 0.5% Oil red O solution (Oil red O saturated solution in isopropanol:water, 3:2, generous gift from Barbieri`s lab, FIU, Miami, USA), followed by repeated water washings. Representative photomicrographs are shown for NCR and HCARDA conditions. Lipid droplets were counted in three different samples of each condition and expressed as number of droplets per cell.

### Immunofluorescence staining

Fixed spermatogenic cells were washed with PBS and permeabilized with 0.1% Triton X-100 (Sigma, T8532) in PBS containing 2% BSA (Fraction V, Sigma, A8022). Then, cells were incubated -away from light- with different markers alone or combined: a primary antibody against α-tubulin (1:50, MP Biomedicals, 691251) to localize the manchette, secondary antibody conjugated with biotin (pan-specific antibody, Vector, PK7800) and avidin-fluorescein complex (Vector, SA5001); antibody against α-actin conjugated with Cy3 (3 μg/ml, Sigma, C6198); Cholera toxin subunit β conjugated with alexa fluor 594 (5 μg/ml, MP Biomedicals, C22842) or with FITC (Sigma, C1655) in order to identify GM1 sphingolipid, a component of lipid rafts; Propidium iodide (1 μg/ml, Sigma, P4864) was applied to identify nuclear material. After washing, cells were mounted with fluoroshield (Sigma, F6182) and examined using inverted microscope NIKON TE2000.

### Testicular efficiency measurement

In both experimental conditions (NCR and HCARDA) it was calculated: a. the percentage of different cell types / total cells; b. indexes for proliferation and differentiation: proliferation efficiency rate (***per***) was calculated dividing the number of ejaculated sperm by the percentage of spermatogonia in the seminiferous tubules calculated above:

$$per=\frac{number\;of\;ejaculated\;sperms}{spermatogonia\;percentage}$$

Differentiation efficiency rate (***der***) was calculated dividing the number of ejaculated sperm by the percentage of spermatids:

$$der=\frac{number\;of\;ejaculated\;sperms}{round+elongated\;spermatids\;percentage}$$

***Per*** and ***der*** indexes for NCR were used as a standard parameter (control condition) and plotted as 100%.

### Apoptosis

Fixed spermatogenic isolated cells were washed with PBS and permeabilized with 0.1% Triton X-100. Detection and quantification of apoptosis (programmed cell death) at single cell level was based on labeling of DNA strand breaks (TUNEL technology, In situ cell death detection kit, 11684795910, Roche). The TUNEL reaction mix was prepared adding 1 part of enzyme solution to 9 parts of label solution. Cells were incubated 60 minutes at 37°C in dark with the TUNEL reaction mixture. After washing, cells were mounted with fluoroshield (Sigma, F6182) and examined using inverted microscope NIKON TE2000.

### Statistical analysis

Unless otherwise expressly noted, results were reported as means ± SEM of at least three independent experiments. Differences between groups were evaluated by ANOVA test, followed by LSD Fisher test, considering a *p* value of less than 0.05 as statistically significant.

## Results

### Serum and semen analyses

Fortnightly serum and semen samples were analyzed. The HCARDA condition presented a significant increase in blood cholesterol (87.5 ± 12.0 mg/dl) compared to NCR (25.2 ± 4.0 mg/dl) after 4 months of the experimental diet (ED). The analyzed semen parameters yielded the same results previously published [4]. Moreover, total sperm count was significantly decreased in semen from HCARDA (478 ± 70 x 10^6^/ejaculate) compared to NCR (225 ± 51 x 10^6^/ejaculate).

### Histological changes observed by light and electron microscopy

#### Light microscopy

**Fig 1:**
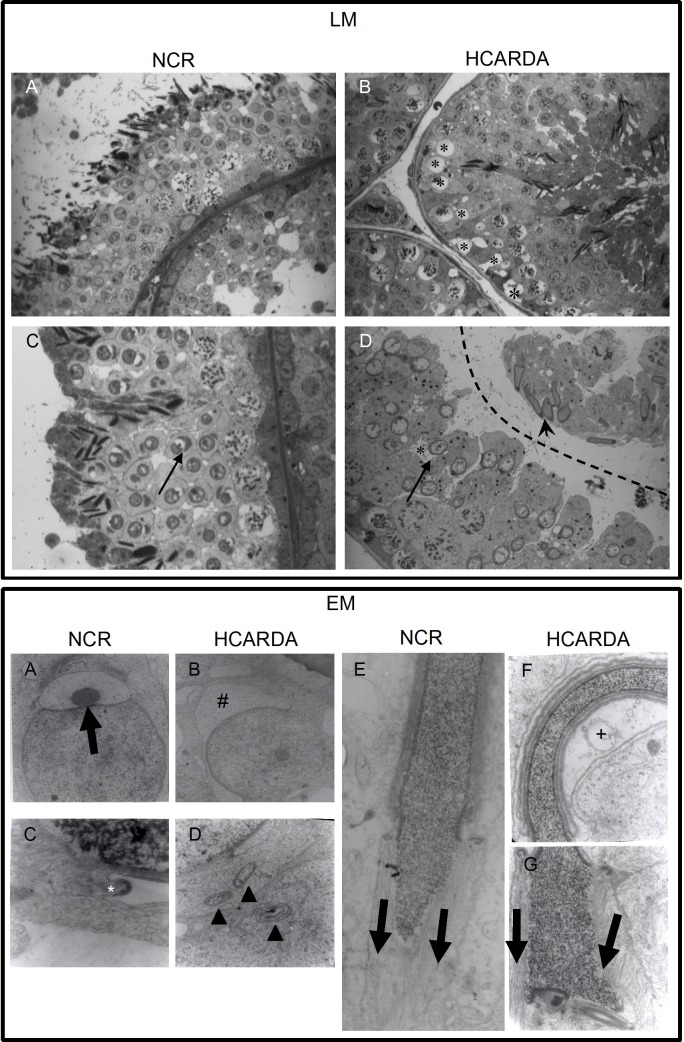
Histological alterations observed by light and electron microscopy. Light Microscopy (LM): Seminiferous tubule cross-sections of NCR (A and C) and HCARDA (B and D). Normal evolution from spermatogonium to sperm cells (A) and distinctive cells from VIII stage (C) are observed. An acrosomic granule is well formed within the Golgi vesicle in NCR(C, arrow). In HCARDA; it was detected: empty holes (lipid droplets, B, asterisks); abnormal development of sperm head (D, arrow head); round spermatids with a big vacuole close to the nucleus and abnormal Golgi features (D, arrow)and elongated spermatids with asymmetric and flexuous nucleus (D, arrowhead). 400X (A and B) and 620X (C and D). Electron Microscopy (EM): Ultrastructure of acrosome development and nucleus shaping of NCR (A, C and E) and HCARDA (B, D, F and G). The acrosomal granule was observed centrally located within the Golgi vesicle (A, arrow). Perinuclear ring of the manchette is indicated (C, asterisk). The microtubule mantle of the manchette was observed parallelly assembled (E, bold parallel arrows) and symmetrically distributed from the central axis (see acrosomal asymmetry measurement in materials and methods). In HCARDA, it was observed: misshapen and asymmetrical proacrosomal vesicle with narrow and expanded zones (B, #); membrane whorls inside spermatogenic cells (D, arrowheads); membranous vacuoles beside the acrosome (F, +); curved sperm heads with non-parallel assembled manchette microtubules (G, unparallel arrows). Magnifications: A, B: 10000X; C, D: 40000X; E, F, G: 20000X.

Spermatogenic cells from different stages in the cycle of the seminiferous epithelium were clearly detected in seminiferous tubules of adult rabbits (Fig 1, LM). Stages V to VIII showed spermatocytes, spermatids (round and elongated) and Golgi-acrosome complex under development inside of round spermatids (Fig 1, LM: C, black arrow). Tissue sections of spermatogenic epithelium in HCARDA showed the presence of empty holes that correspond to lipid droplets extracted during dehydration of tissue in preparation for light microscopy (Fig 1, LM: B, asterisks). Animals under HC showed several alterations: the seminiferous epithelium was detached (Fig 1, LM: D, dashed line); round spermatids contained lipid vacuoles near to the Golgi apparatus (Fig 1, LM: D, arrow points the cell and asterisk the vacuole) and elongated spermatids displayed nucleus with abnormal morphology (Fig 1, LM: D, arrow head).

#### Electron microscopy

Acrosome development in NCR was detected by the formation of a single, large acrosomal granule within a larger vesicle that indents the nucleus. Acrosomal granule was centrally located, equidistantly from both proacrosomal vesicle edges (Fig 1, EM: A, black arrow). During the acrosomal development, a peri-nuclear ring was observed around the nucleus, delimiting the proximal groove of acrosome (Fig 1C, asterisk). Instead, in HCARDA, the acrosomal vesicle showed asymmetrical and both narrow and expanded in certain areas (Fig 1, EM: B, #). Moreover, membrane whorls previously described in cells under hypercholesterolemic conditions [24] were also noted (Fig 1, EM: D, arrowheads). In NCR, the manchette microtubules were observed to be symmetrically pulling towards the future sperm tail (Fig 1, EM: E, parallel black arrows). This microtubular arrangement in HCARDA was also observed, but some sperm heads appeared curved indicating an asymmetrical assembly (Fig 1, EM: F, G, non-parallel black arrows). Membranous vacuoles in the proximity of the acrosome were also observed (Fig 1, EM: F, +).

### Sperm morphological alterations

**Fig 2:**
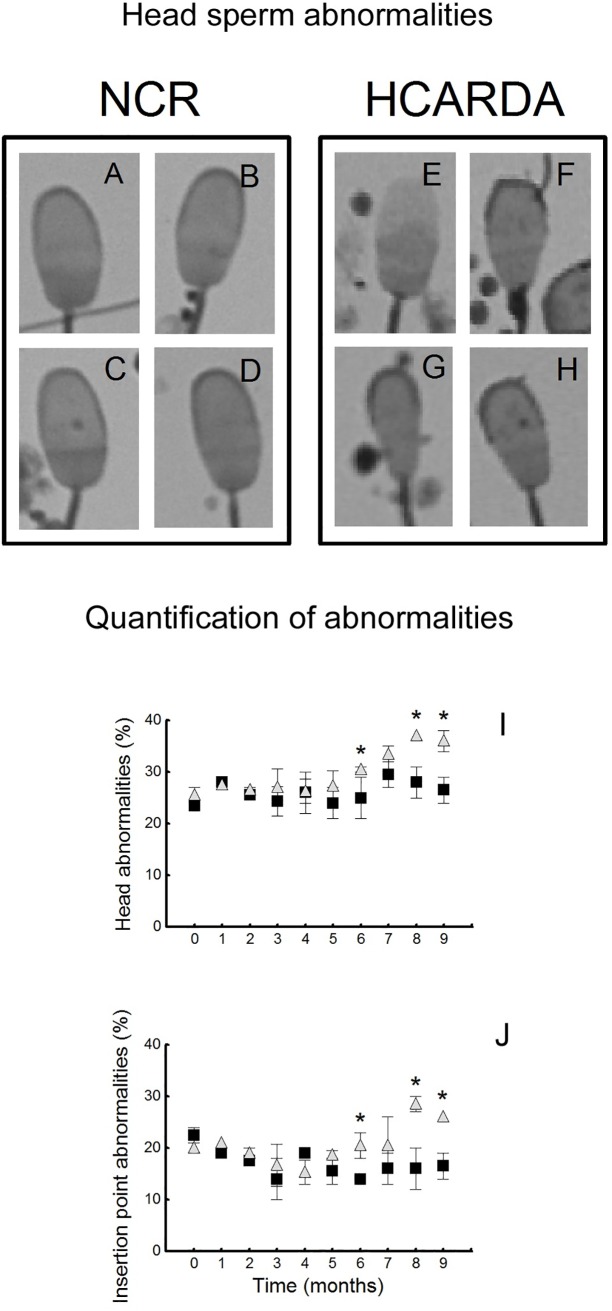
Morphological changes in seminal sperm. Representative sperm cells isolated from semen of NCR (left panel) and HCARDA (right panel) rabbits. Four sperm heads from NCR show the slight variance among the normal sperm population found (A, B, C, D). Instead, several abnormalities were observed in HCARDA: acrosomal lost (E), cytoplasmic droplet persistence (F), tapered head (G) and asymmetry in the implantation of the tail (H). 1000X. Cells with head and tail defects from NCR (■) and HCARDA (Δ) were quantified and are represented as the mean ± SD of the ratio between the number of alterations / 100 sperm cells counted in thirty different cells. The experimental time is represented in x axis since the beginning of ED. Percentages were significantly different (*p* ≤ 0.05) from six months of ED.

Seminal sperm cells stained with Giemsa were evaluated in order to assess morphological abnormalities. Several sperm head alterations were found in NCR (Fig 2A, 2B, 2C and 2D) and in HCARDA (Fig 2E, 2F, 2G and 2H): spontaneous acrosome lost, abnormal acrosome shape, cytoplasmic droplet persistence, elongated heads and asymmetric insertion of the tail. Sperm cells with head and flagellum insertion alterations were tabulated and expressed as a percentage of total cell count (Fig 2I and 2J). Differences between NCR and HCARDA were significant at 6 months of ED consumption.

### Spermatogenic cells isolation and acrosomal asymmetry: Asymmetry index

**Fig 3:**
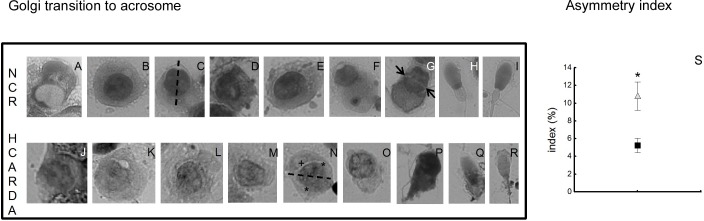
Morphological alterations in isolated spermatogenic cells and acrosomal asymmetry (Asymmetry Index). Isolated cells were arranged from round (left) to elongated spermatids (right) following successive steps of transition from Golgi to acrosome in NCR (A to I, upper row) and HCARDA (J to R, lower row). Dashed line denotes the central axis(C and N); asterisks indicate acrosomal ends (N); arrow points the nuclear ring position (G) and abnormal vacuole is marked with + (N). 650X. **Asymmetry Index** (S): Distance from the central axis to each acrosomal end in isolated cells (*n* = 30 cells per condition) was calculated and expressed as asymmetry index (NCR: ■, HCARDA: Δ). A major index corresponds to higher asymmetry (*p* ≤ 0.003).

Isolated spermatogenic cells stained with Toluidine blue were randomly observed and photographed from NCR and HCARDA buds. The symmetry of acrosomal development was evaluated by measuring the distance from the imaginary central axis to each acrosomal edge (S1 Fig), compared to the distance between both acrosomal ends. Fig 3 shows spermatogenic cells ordered by stages of development [25] (A to I for NCR and J to R for HCARDA). The nuclear material condenses in successive steps while elongation occurs (Fig 3A to 3I, left to right). In control animals, it was observed: the central axis dividing the nucleus as a mirror image (Fig 3C, dashed line), the peri-nuclear ring position ahead of the manchette complex (Fig 3G, arrows) and absence of big membranous vacuoles. Instead, in HCARDA, main alterations were: misshapen heads, abnormal acrosome development (asymmetrically regarding the central axis, Fig 3N), presence of big vacuoles alongside the acrosome (Fig 3N, +) and inaccurate location of the imaginary central axis (Fig 3N, dashed line). Distances from central axis to each acrosomal end were measured by Image J software and plotted as an index (see Material and Methods, Asymmetry Index). The Asymmetry Index was significantly different between NCR and HCARDA (Fig 3S).

### Lipid storage—ORO staining

The impact of ED on neutral lipid storages in spermatogenic cells was studied. Stained droplets with ORO reagent inside cells were observed and counted. Cells from HCARDA displayed a significant increase of lipid droplets compared to NCR (S2 Fig).

### Abnormal head development during spermiogenesis

**Fig 4:**
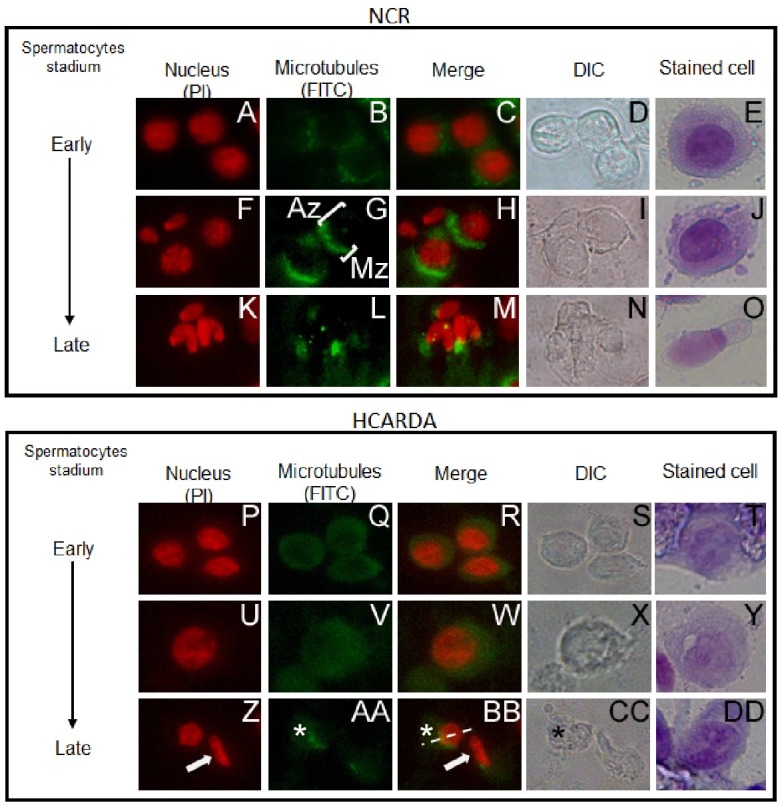
Manchette organization during spermiogenesis. Spermatogenic cells were isolated, stained and ordered according acrosome maturation (Early to Late). Spermatid nuclei were stained red with propidium iodide (A, F, K, P, U, Z) and manchette microtubules were visualized by anti-Alpha tubulin (green, B, G, L, Q, V, AA). In NCR, it was visualized: a polarized manchette (G, Mz: Manchette zone, H) in opposite location to the acrosome (G, Az: Acrosome zone, H) and condensed nuclei (K, M). Instead, a diffused manchette (Q, V, AA), abnormal nucleus condensation (P, U, Z, white arrow) and persistency of residual bodies (AA, BB, CC, asterisks) were visualized in HCARDA. Dashed line indicates the central axis. Phase-contrast microscopy images of the corresponding immunofluorescence images are included (DIC). Last column shows stained cells resembling the same stadium. *n* = 100 cells. Magnification:650X.

Manchette complex assembly in spermatogenic cells was detected and traced. The transformation of spherical spermatids (Early) into elongated, highly condensed and mature spermatozoa (Late) involved regular nuclear condensation to achieve a palette shaped head (Fig 4, NCR: A, F, K and E, J, O). Some nucleus from HCARDA showed abnormal elongation and condensation (45% HCARDA *vs*. 16% NCR; Fig 4, HCARDA: P, U, Z and T, Y, DD). Anti alpha-tubulin stains the manchette complex. In cells from NCR, it was observed an homogeneous distribution of tubulin around the nucleus (Fig 4, NCR: B) which gradually ended up being located in an area related to the growing flagellum (Fig 4, NCR: G). Finally, the manchette disassembled upon nuclear elongation and was observed as a shadow moving with the residual body (Fig 4, NCR: L). During spermiogenesis, manchette organization displayed altered in HCARDA (52%) compared to NCR (19%, Fig 4, compare NCR panel with HCARDA panel). In this condition, alpha-tubulin was not primarily located at the corresponding nuclear pole, and showed less intense and diffused (Fig 4, HCARDA: Q, V, AA). Moreover, cells from HCARDA showed not only an abnormal homogeneous allocation of microtubules around the nucleus but also an asymmetric distribution from the central axis (Fig 4, HCARDA: AA, BB, dashed line indicates the central axis). The altered manchette architecture was also accompanied by abnormal nuclear shaping and non-homogeneous nuclear material in cells from HCARDA (Figs 2E–2H; 3P–3R; 4, HCARDA: Z).

### Lipid rafts and cytoskeleton (microtubules of manchette and actin filaments) during spermiogenesis

**Fig 5:**
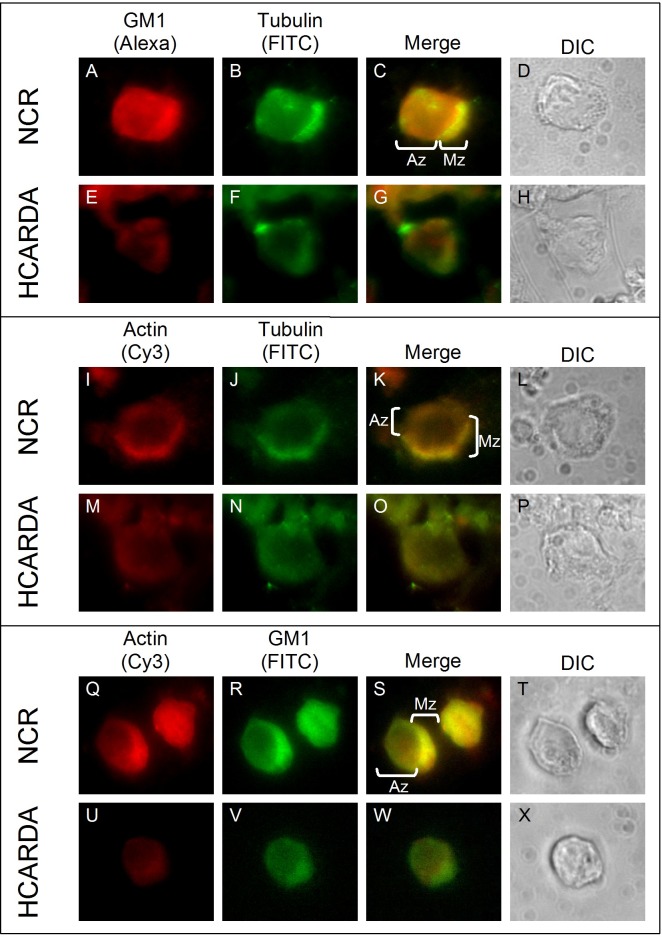
Manchette (microtubules), GM1 (raft membrane micro domains) and actin filaments arrangement during spermiogenesis. Spermatogenic cells were isolated, stained and observed under fluorescence microscopy. Manchette microtubules were detected with anti alpha-tubulin (green, B, F, J, N), actin filaments were localized with anti alpha-actin (red, I, M, Q, U) and GM1 sphingolipids were detected by cholera toxin beta subunit (red, A, E; green, R, V). In control cells, the manchette was polarized (NCR; B, J) and co-localized with GM1 (NCR; C—Mz), opposite to the acrosome (K, Az). Instead, alpha-tubulin and GM1 were diffused and without co-localization in cells isolated from HCARDA (E, F, G). Actin filaments were localized with alpha-tubulin in the manchette (I, J, K—Mz) in NCR. But in HCARDA, actin and tubulin were visualized diffused (M, N, O). Interestingly, actin and GM1 were localized in the manchette in NCR (Q, R, S—Mz) but were seen dispersed in HCARDA (U, V, W). Phase-contrast microscopy images of the corresponding immunofluorescence images are included (DIC). Last column shows stained cells resembling the same stadium. Mz: manchette zone; Az: acrosomal zone. *n* = 100 cells. Magnification: 650X.

Manchette microtubules, actin filaments and GM1 ganglioside enriched lipid rafts distribution was studied in spermatogenic cells for the first time. As it was described above, the manchette was polarized in NCR condition, but appeared diffused in HCARDA (Fig 5B, 5F, 5J and 5N, green). In NCR, an enriched GM1 signal (raft) was located in the post-nuclear area related with the future flagellar growth region (Fig 5A, red). Microtubules and GM1 co-localized in NCR (Fig 5C, Merge). On the contrary, in HCARDA both GM1 and alpha-tubulin were visualized diffused in the perinuclear area, without co-localization inside abnormal shaped spermatids (Fig 5E, 5F and 5G). Actin filaments were localized mainly in the manchette in NCR (Fig 5I, red). Remarkably, microtubules displayed the same distribution (Fig 5J, green; 5K, merge). Instead, in HCARDA actin and alpha tubulin displayed dispersed and diffused throughout the spermatids cytoplasm (Fig 5M, 5N and 5O). Actin filaments also co-localized with GM1 (raft) in the post-nuclear area in NCR, as it was expected (Fig 5Q, 5R and 5S). In HCARDA condition, actin and alpha tubulin were visualized diffused throughout the cytoplasm (Fig 5U, 5V and 5W).

### Testicular efficiency measurement

**Fig 6:**
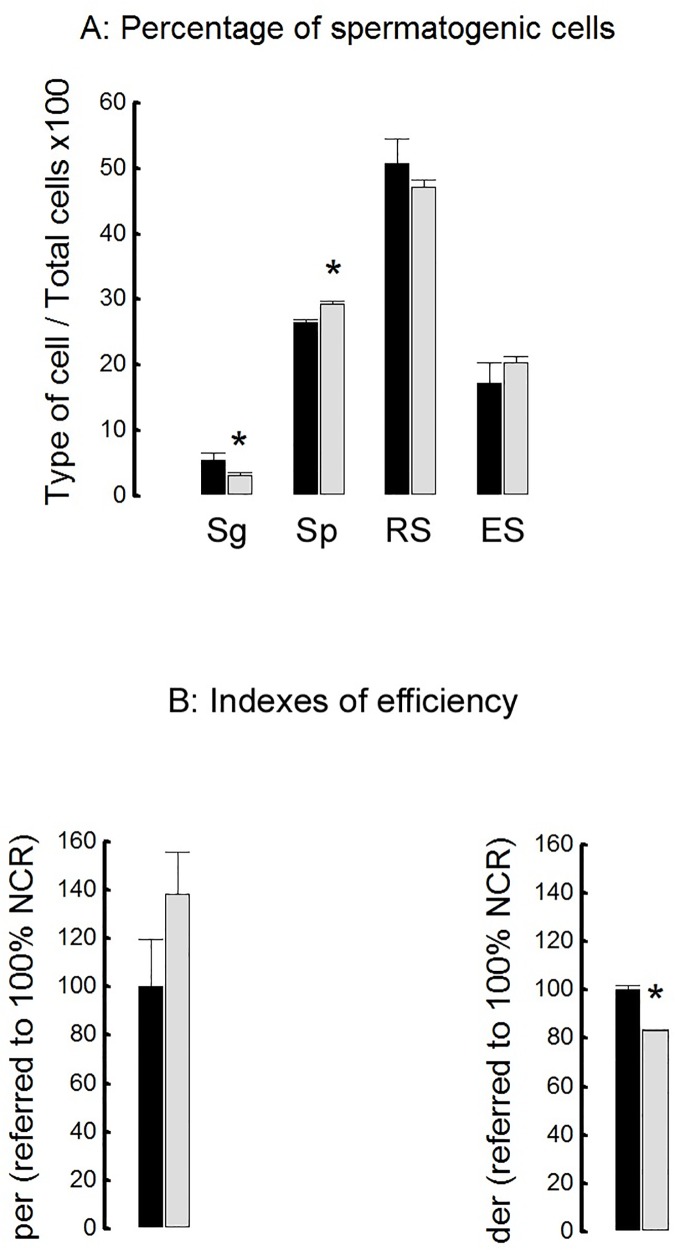
Testicular efficiency. A: Percentage of spermatogenic cells. Sg: Spermatogonia; Sp: Spermatocytes; RS: Round Spermatids; ES: Elongated Spermadids. Mean ± SD of percentages of different spermatogenic cells was plotted for both conditions (NCR = black bars; HCARDA = grey bars). * *p*<0.05. B: Proliferation efficiency rate (***per***) and differentiation efficiency rate (***der)*** were normalized for NCR. Both indexes were calculated in three separated experiments. Mean ± SD of ***per*** and ***der*** (NCR = black bars; HCARDA = grey bars) was plotted. *n* = 200 cells per condition. * *p*<0.05. Apoptosis was tested in isolated cells and we found a dramatic increase in germ cell apoptosis in HCARDA rabbits (21%) compared to NCR (9%).

Efficiency of spermatogenesis was calculated as the number of spermatogenic cells per cross-section. Hypercholesterolemic rabbits (HCARDA) displayed significant low percentage of spermatogonial cells (Sg), but higher number of spermatocytes (Sp) compared to normal cholesterolemic rabbits (NCR, Fig 6, l A). No significant differences between both experimental groups were detected in the percentage of round (RS) and elongated (ES) spermatids (Fig 6A). Indexes for proliferation and differentiation were estimated. Proliferation efficiency rate (***per***) between both groups was not statistically significant but, differentiation efficiency rate (***der)*** was significantly lower in HCARDA compared to NCR (Fig 6B).

## Discussion

This paper describes the effects of a high cholesterol diet on sperm number and morphology during spermatogenesis in rabbits. The decrease in semen sperm number could be caused by germ cell apoptosis. Abnormal sperm morphology could be explained by manchette disorganization that involves lipid rafts. Thus, this paper reports -for the first time-sperm cholesterol content linked to morphological disorders.

Several animal and clinical studies have been carried out to focus on the association of hypercholesterolemia with male infertility (Reviewed in [26]). Experimental studies performed in Hypercholesterolemic animal models display detrimental effect of high cholesterol diets on the structure and functions of testes and accessory sex organs, epididymal sperm maturation, sperm quality parameters and sperm fertilizing capacity [4, 26–35]. We have previously reported a decreased semen quality in hypercholesterolemic rabbits [4] that can be improved by olive oil addition to the grease diet [36]. Moreover, previous studies have shown that cholesterol-enriched diets affect sperm fertilizing capacity and embryonic development in this specie [30].

One of the main sperm modifications triggered by hypercholesterolemia is higher cholesterol content in sperm cells. Some cells accumulate excess of cholesterol as lipid droplets [24]. Tissue sections of spermatogenic epithelium in HCARDA showed the presence of empty holes that probably corresponds to lipid droplets extracted during dehydration of tissue in preparation for light microscopy. We also found increased lipid storages in isolated spermatogenic cells from HCARDA compared to control. Interestingly, ultrastructural studies showed the presence of membrane whorls inside spermatogenic cells, previously described as intracellular cholesterol overload [24]. The presence of vacuoles has been previously described in normal rabbits [37], but the cell type containing these vacuoles was Sertoli cells. Instead, we found lipid droplets and whorls inside spermatogenic cells. These observations demonstrate that fat diet has direct impact on lipid / cholesterol increment in testis cells.

Abnormal sperm morphology was analyzed using different techniques to define the mechanisms involved. Under light microscopy, some abnormal elongated spermatid cells and Golgi–acrosome complex alterations were detected during the process of spermiogenesis. These morphological changes moved us to ultrastructural analyses. Dysmorphic acrosomal development resulted in abnormal sperm head. This atypical acrosome was accompanied also by abnormal nuclear shaping and elongation. Acrosome and nucleus development depends on manchette -a microtubular hair- that normally pulls symmetrically from a perinuclear ring towards the future sperm tail [10, 23]. A large percentage of cells from HCARDA (52%) presented alterations in the microtubular arrangement, showing a non parallel structure. As a consequence, curved sperm heads were produced and detected. This mechanism could lead to folded sperm heads similar to the ones reported previously [38]. On the other hand, there is growing research indicating the relationship between the processes governing the development of the manchette and the sperm tail [38]. The asymmetry in the flagellar position in HCARDA could be a side effect of acrosome-manchette asymmetry. Supporting this, the shedding of the residual body toward the sperm tail was detected away from the central axis in HCARDA. Taken together, these changes could indicate that lipid excess influences sperm morphology.

The association between cytoskeletal components and lipid rafts had been previously described [39, 40]. Recent data proves that cytoskeletal components can localize to lipid rafts and be platforms for cytoskeletal tethering [41, 42]. Moreover, rafts can cluster depending upon cholesterol and actin tethering to the membrane [43]. We propose that the same scenario could be present in spermatids while acquiring the final sperm shape, a cytoskeleton dependent process. Therefore, we enquired whether cholesterol enrichment of rafts might alter the organization and location of microtubules and actin filaments during manchette development. As it was demonstrated in Figs 4 and 5, the three main cell elements studied here (actin, microtubules and GM1) are involved in the accurate assembly of the manchette complex. We found that GM1 sphingolipid was dispersed throughout the membrane in spermatids from HCARDA, instead of been polarized (as in NCR). Both actin and alpha-tubulin co-localized with polarized raft microdomains in NCR, but appeared scattered (following GM1 distribution) in HCARDA. The latter supports the idea of a disassembly of the manchette structure into a diffused, non polarized pattern.

We have previously demonstrated that fat diet promoted cholesterol enrichment on sperm membrane in White New Zealand rabbits. The decrease in membrane fluidity due to cholesterol enrichment may impair raft mobility inside the membrane, thereby preventing rearrangement and aggregation [16]. Thus, interfering with raft coupled functions: capacitation, RA [4] and also association with cytoskeletal components. Taken together, accurate concentration of cholesterol in sphingolipid-rich microdomains are essential for actin and microtubule-based manchette assembly. So far it is not clear which molecular machinery is involved in the interaction of acroplaxomal-manchette complex with lipid microdomains.

The measurement of sperm count or concentration has long been one of the most feasible approaches for human semen evaluation [44], and may be a sensitive indicator of reproductive function. Some authors have reported a significant reduction in sperm concentration in different animal models of hypercholesterolemia (reviewed in [26]). Contrary to this, we showed no relation between cholesterolemia and sperm concentration. This could be due to the high variability of rabbit sperm production due to environmental factors [45]. Some factors were controlled as the rank of the ejaculate (it was always collected the first ejaculate), human factor (same technician in charge of semen collection), and age of bucks. However, other factors that were shown to affect semen characteristics as the season of collection were not taken into account. Nevertheless, we found a significant reduction in total sperm count in animals under fat diet.

Testicular efficiency was established as the relation between testis weight and ejaculated sperm number [46]. But in our experimental conditions testicular weight did not significantly decrease as it was reported by other authors (data not shown, [30]). However, cholesterol feeding produced a marked diminution in the spermatogenic cell population. The production of spermatogonial cells was reduced by 56%.

Proliferation rate did not show a significative change between rabbits of both conditions, because both spermatogonial cells and ejaculated sperm cells were reduced. On the other hand, differentiation rate was lower for HCARDA, as the number of spermatid cells was similar in both experimental groups. It is possible that the developing cells reach the differentiation compartment and then both normal and altered cells may be released (spermiation) toward the lumen of the seminiferous tubule. These results are supported by an increment of abnormal ejaculated sperm. These results taken together demonstrate that in HCARDA the number of spermatogonia is lower despite proliferation rate does not change, and differentiation rate is compromised in spite of the unchanged number of spermatids.

Apoptosis occurs at a high rate in the primary male reproductive organ, the testis. [47]. Spermatogenesis is normally accompanied by germ cell apoptosis in the seminiferous epithelium and apoptosis here could be triggered by a variety of stimuli [48, 49]. We found increased germ cell apoptosis in isolated cells from HCARDA that explains directly the low ejaculated sperm number.

## Conclusions

Hypercholesterolemia induces detrimental effects on spermatogenesis that lead to low sperm number and abnormal sperm morphology in rabbit’s semen. The decline in testicular efficiency is related to a decrease in spermatogonia and an increase in germ cell apoptosis. Sperm defects are a result of a defective interaction between the acrosome-manchette complex and membrane microdomains. Thus, on the basis of above results and evidence in humans [50, 51, 52, 53], it can be proposed that hypercholesterolemia is an important factor contributing to male infertility. Therefore, the correlation between semen parameters and serum cholesterol level may help in diagnosis and treatment of male infertility, especially in idiopathic cases.

## Supporting information

S1 Fig Acrosomal Asymmetry Index. A: Spermatogenic cells isolated from seminiferous tubules. Dashed lines indicate the central axis and numbers 1 and 2 the distances from each acrosomal end (Left column). Note the presence of a vacuole in the spermatid´s cytoplasm of HCARDA (*), displacing the acrosomal granule (black arrow). 650X. B: Diagrammatic representation of an elongated spermatid. The microtubule-containing manchette (manchette zone = Mz) is inserted in a perinuclear ring to produce the stretching of acrosome around the nucleus (acrosomal zone = Az).
(TIF)

S2 Fig Lipid (Oil red O) staining and quantification of lipid droplets. A—H: Isolated cells from seminiferous tubules showing neutral lipids stained with ORO. A–D: NCR; E–G: HCARDA. Bars represent 50 μm. Right column corresponds to magnification of some positive cells (B, D, F, G). I: Quantification of lipid droplets inside spermatogenic cells from NCR (■, 0.886 ± 0.331) and HCARDA (Δ, 4.158 ± 1.808) of 5 different experiments (Mean ± SD* = *p* ≤ 0. 01).
(TIF)

## References

[1] WHO. Serie de Informes Técnicos 916. DIETA, NUTRICIÓN Y PREVENCIÓN DE ENFERMEDADES CRÓNICAS http://www.who.int/nutrition/publications/obesity/WHO_TRS_916_spa.pdf).

[2] Whitfield M, Pollet-Villard X, Levy R, Drevet JR, Saez F. Posttesticular sperm maturation, infertility, and hypercholesterolemia. Asian J Androl. 2015. [Epub ahead of print] 10.4103/1008-682X.155536 PMC4577583 26067871

[3] Kasturi SS, Tannir J, Brannigan RE. The metabolic syndrome and male infertility. J Androl 2008; 29: 251–9. 10.2164/jandrol.107.003731 18222914

[4] Saez Lancellotti TE, Boarelli PV, Monclus MA, Cabrillana ME, Clementi MA, Espinola LS, et al Hypercholesterolemia Impaired Sperm Functionality in Rabbits. PLoS ONE 2010; 5(10): e13457 10.1371/journal.pone.0013457 20976152 PMC2956674

[5] Marchiani S, Vignozzi L, Filippi S, Gurrieri B, Comeglio P, Morelli A, et al Metabolic syndrome-associated sperm alterations in an experimental rabbit model: relation with metabolic profile, testis and epididymis gene expression and effect of tamoxifen treatment. Mol Cell Endocrinol. 2015; 401: 12–24. 10.1016/j.mce.2014.11.005 25451982

[6] Johnson L. Efficiency of spermatogenesis. Microsc Res Tech. 1995; 32(5):385–422. 10.1002/jemt.1070320504 8563040

[7] França LR, Godinho CL. Testis morphometry, seminiferous epithelium cycle length, and daily sperm production in domestic cats (Feliscatus). Biol Reprod. 2003; 68(5):1554–61. 10.1095/biolreprod.102.010652 12606460

[8] Abdou MS, Hassun TM, El-Sawaf SA. Testicular and epididymal sperm numbers and related parameters in the developing awassiram. Aust J Biol Sci. 1978; 31(3):257–66. 727994 10.1071/bi9780257

[9] Chemes HE, Rawe VY. The making of abnormal spermatozoa: cellular and molecular mechanisms underlying pathological spermiogenesis. Cell Tissue Res.2010; 341(3):349–57. Epub 2010 Jul 2. 10.1007/s00441-010-1007-3 20596874

[10] Kierszenbaum AL, Tres LL. The acrosome-acroplaxome-manchette complex and the shaping of the spermatid head. Arch Histol Cytol. 2004; 67: 271–284. 15700535 10.1679/aohc.67.271

[11] Lehti MS, Sironen A. Formation and function of the manchette and flagellum during spermatogenesis. Reproduction. 2016; 151(4):R43–54. 10.1530/REP-15-0310 26792866

[12] Chini B and Parenti M. G-protein coupled receptors in lipid rafts and caveolae: how, when and why do they go there? J. Mol. Endocrinol. 2004; 32: 325–348. 15072542 10.1677/jme.0.0320325

[13] Head BP, Patel HH, Roth DM, Murray F, Swaney JS, Niesman IR, et al Microtubules and actin microfilaments regulate lipid raft/caveolae localization of adenylyl cyclase signaling components. The Journal of biological chemistry. 2006; 281:26391–26399. [PubMed: 16818493] 10.1074/jbc.M602577200 16818493

[14] Suzuki T, Zhang J, Miyazawa S, Liu Q, Farzan MR, Yao WD. Association of membrane rafts and postsynaptic density: proteomics, biochemical, and ultrastructural analyses. Journal of neurochemistry. 2011; 119:64–77. [PubMed: 21797867] 10.1111/j.1471-4159.2011.07404.x 21797867 PMC3184177

[15] Kabouridis PS, Janzen J, Magee AL and Ley SC. Cholesterol depletion disrupts lipid rafts and modulates the activity of multiple signaling pathways in T lymphocytes. Eur. J. Immunol. 2000; 30: 954–963. 10.1002/1521-4141(200003)30:3<954::AID-IMMU954>3.0.CO;2-Y 10741414

[16] Larbi A, Douziech N, Dupuis G, Khalil A, Pelletier H, Guerard KP, et al Age-associated alterations in the recruitment of signal-transduction proteins to lipid rafts in human T lymphocytes. J Leukoc Biol. 2004; 75(2):373–81. 10.1189/jlb.0703319 14657209

[17] Kannan KB, Barlos D, Hauser CJ. Free cholesterol alters lipid raft structure and function regulating neutrophil Ca2+ entry and respiratory burst: correlations with calcium channel raft trafficking. J Immunol. 2007; 178(8):5253–61. 17404309 10.4049/jimmunol.178.8.5253

[18] Bredderman PJ, Foote RN, Yassen AM. An improved artificial vagina for collecting rabbit semen. Journal of Reproduction and Fertility 1964; 7: 401–403. 14180733 10.1530/jrf.0.0070401

[19] Orgebin-Crist MC. Studies on the function of the Epididymis. Biology of Reproduction 1969; 1: 155–175. 10.1095/biolreprod1.supplement_1.155 5406325

[20] Mollenhauer HH, Hass BS, Morre DJ. Membrane transformation in Golgi apparatus of rat spermatids. A role for thick cisternae and two classes of coated vesicles in acrosome formation. J Microsc Electron Biol. Celular 1976; 27: 33–36.

[21] Swierstra EE, Foote RH. Cytology and kinetics of spermatogenesis in the rabbit. J ReprodFertil. 1963; 5, 309–322 10.1530/jrf.0.0050309 13979709

[22] Parvinen LM, Söderström KO, Parvinen M. Early effects of vinblastine and vincristine on the rat spermatogenesis: analyses by a new transillumination-phase contrast microscopic method. Exp Pathol (Jena) 1978; 15(2):85–96. 354959 10.1016/s0014-4908(78)80072-6

[23] Kierszenbaum AL, Rivkin E, Tres LL. Acroplaxome, an F-actin-keratin-containing plate, anchors the acrosome to the nucleus during shaping of the spermatid head. Mol Biol Cell. 2003; 14(11):4628–40. 10.1091/mbc.E03-04-0226 14551252 PMC266778

[24] Tabas I. Consequences of cellular cholesterol accumulation: basic concepts and physiological implications. J. Clin. Invest. 2002; 110:905–911 10.1172/JCI16452 12370266 PMC151158

[25] Berndston WE, Desjardins C. The cycle of the seminiferous epithelium and spermatogenesis in the bovine testis. Am J Anat. 1974;140(2):167–79. 10.1002/aja.1001400204 4826242

[26] Pushpendra A, Jain GC. Hyper-Lipidemia and Male Fertility: A Critical Review of Literature. Andrology (Los Angel) 2015; 4:141.

[27] Gupta RS, Dixit VP. Effect of dietary cholesterol on spermatogenesis. Z Ernahrungswiss 1988; 27: 236–243. 3239111 10.1007/BF02019512

[28] Shimamoto K, Sofikitis N. Effect of hypercholesterolaemia on testicular function and sperm physiology. Yonago Acta Medica 1988; 41: 23–29.

[29] Purohit A, Daradka HM. Effect of mild hyperlipidaemia on testicular cell population dynamics in albino rats. Indian J Exp Biol. 1999; 37: 396–398. 10641176

[30] Yamamoto Y, Shimamoto K, Sofikitis N, Miyagawa I. Effects of hypercholesterolaemia on Leydig and Sertoli cell secretory function and the overall sperm fertilizing capacity in the rabbit. Hum Reprod. 1999; 14: 1516–1521. 10357968 10.1093/humrep/14.6.1516

[31] Tanaka M, Nakaya S, Kumai T, Watanabe M, Matsumoto N, Kobayashi S. Impaired testicular function in rats with diet-induced hypercholesterolemia and/or streptozotocin-induced diabetes mellitus. Endocr Res. 2001; 27: 109–117. 11428703 10.1081/erc-100107174

[32] Shalaby MA, el-Zorba HY, Kamel GM. Effect of alpha-tocopherol and simvastatin on male fertility in hypercholesterolemic rats. Pharmacol Res. 2004; 50: 137–142. 10.1016/j.phrs.2003.10.013 15177301

[33] Bataineh HN, Nusier MK. Effect of cholesterol diet on reproductive function in male albino rats. Saudi Med J. 2005; 26: 398–404. 15806207

[34] Ouvrier A, Alves G, Damon-Soubeyrand C, Marceau G, Cadet R, Janny L, et al Dietary cholesterol-induced post-testicular infertility. PLoS One 2011; 6: e26966 10.1371/journal.pone.0026966 22073227 PMC3206870

[35] Ashrafi H, Ghabili K, Alihemmati A, Jouyban A, Shoja MM, Aslanabadi S, et al The effect of quince leaf (Cydonia oblonga Miller) decoction on testes in hypercholesterolemic rabbits: a pilot study. Afr J Tradit Complement Altern Med. 2013; 10: 277–282. 24146451 10.4314/ajtcam.v10i2.12 PMC3746575

[36] Saez Lancellotti TE, Boarelli PV, Romero AA, Funes AK, Cid-Barria M, Cabrillana ME, et al Semen Quality and Sperm Function Loss by Hypercholesterolemic Diet Was Recovered by Addition of Olive Oil to Diet in Rabbit. PLoS ONE 2013; 8(1): e52386 10.1371/journal.pone.0052386 23326331 PMC3543415

[37] Morton D, Weisbrode SE, Wyder WE, Maurer JK, Capen CC. Histologic Alterations in the Testes of Laboratory Rabbits. Vet. Pathol. 1986; 23:2 14–2 17. 10.1177/030098588602300221 3962093

[38] Kierszenbaum AL. Intramanchette transport (IMT): managing the making of the spermatid head, centrosome, and tail. Mol Reprod Dev. 2002; 63: 1–4. 10.1002/mrd.10179 12211054

[39] Viola A, Gupta N. Tether and trap: regulation of membrane-raft dynamics by actin-binding proteins. Nature reviews. Immunology. 2007; 7:889–896. 10.1038/nri2193 17948020

[40] Simons K, Gerl MJ. Revitalizing membrane rafts: new tools and insights. Nature reviews. Molecular cell biology. 2010; 11:688–699. 10.1038/nrm2977 20861879

[41] Whitehead SN, Gangaraju S, Aylsworth A, Hou ST. Membrane raft disruption results in neuritic retraction prior to neuronal death in cortical neurons. Bioscience trends. 2012; 6:183–191. [PubMed: 23006965] 23006965 10.5582/bst.2012.v6.4.183

[42] Goudenege S, Dargelos E, Claverol S, Bonneu M, Cottin P, Poussard S. Comparative proteomic analysis of myotube caveolae after milli-calpain deregulation. Proteomics. 2007; 7:3289–3298. [PubMed: 17849407] 10.1002/pmic.200700124 17849407

[43] Goswami D, Gowrishankar K, Bilgrami S, Ghosh S, Raghupathy R, Chadda R, et al Nanoclusters of GPI-anchored proteins are formed by cortical actin-driven activity. Cell. 2008; 135:1085–1097. [PubMed: 19070578] 10.1016/j.cell.2008.11.032 19070578 PMC7616455

[44] World Health Organization. 2010 WHO Laboratory Manual for the Examination and Processing of Human Semen, 5th edition Geneva, Switzerland: WHO Press, World Health Organization.

[45] Theau-Clement M, Bolet G, Sanchez A, Saleil G, Brun JM. Some factors that influence semen characteristics in rabbits. Animal Reproduction Science 2015; 157: 33–38. 10.1016/j.anireprosci.2015.03.011 25862381

[46] Guan Y, Malecki IA, Hawken PA, Linden MD, Martin GB. Under-nutrition reduces spermatogenic efficiency and sperm velocity, and increases sperm DNA damage in sexually mature male sheep. Anim Reprod Sci. 2014; 149(3–4):163–72. 10.1016/j.anireprosci.2014.07.014 25086661

[47] Shukla KK, Mahdi AA, Rajender S. Apoptosis, spermatogenesis and male infertility. Frontiers in Bioscience 2012; E4, 746–754. 10.2741/415 22201910

[48] Sinha Hikim AP, Lue Y, Diaz-Romero M, Yen PH, Wang C, Swerdloff RS. Deciphering the pathways of germ cell apoptosis in the testis. J Steroid Biochem Mol Biol. 2003; 85, 175–182. 12943702 10.1016/s0960-0760(03)00193-6

[49] Martincic DS, Virant Klun I, Zorn B, Vrtovec HM. Germ cell apoptosis in the human testis. Pflugers Arch. 2001; 442, 59–60. 10.1007/s004240100007 11678322

[50] Rosety I, Elosegui S, Pery MT, Fornieles G, Rosety JM, Díaz AJ, et al Association between abdominal obesity and seminal oxidative damage in adults with metabolicsyndrome. Rev Med Chil. 2014; 142(6):732–7. 10.4067/S0034-98872014000600007 25327318

[51] Leisegang K, Udodong A, Bouic PJ, Henkel RR. Effect of the metabolic syndrome on male reproductive function: a case-controlled pilot study. Andrologia 2014; 46(2):167–76. Epub 2012 Dec 28. 10.1111/and.12060 23278477

[52] Michalakis K, Mintziori G, Kaprara A, Tarlatzis BC, Goulis DG. The complex interaction between obesity, metabolic syndrome and reproductive axis: a narrative review. Metabolism 2013; 62(4):457–78. Epub 2012 Sep 20. 10.1016/j.metabol.2012.08.012 22999785

[53] Kasturi SS, Tannir J, Brannigan RE. The metabolic syndrome and male infertility. J Androl. 2008;29(3):251–9. Epub 2008 Jan 24. 10.2164/jandrol.107.003731 18222914

