# Supplementary figures and images for: Manchette-acrosome disorders during spermiogenesis and low efficiency of seminiferous tubules in hypercholesterolemic rabbit model
Source: PLoS One. 2017 Feb 27;12(2):e0172994. doi: 10.1371/journal.pone.0172994 (PMC5328279; doi:10.1371/journal.pone.0172994)

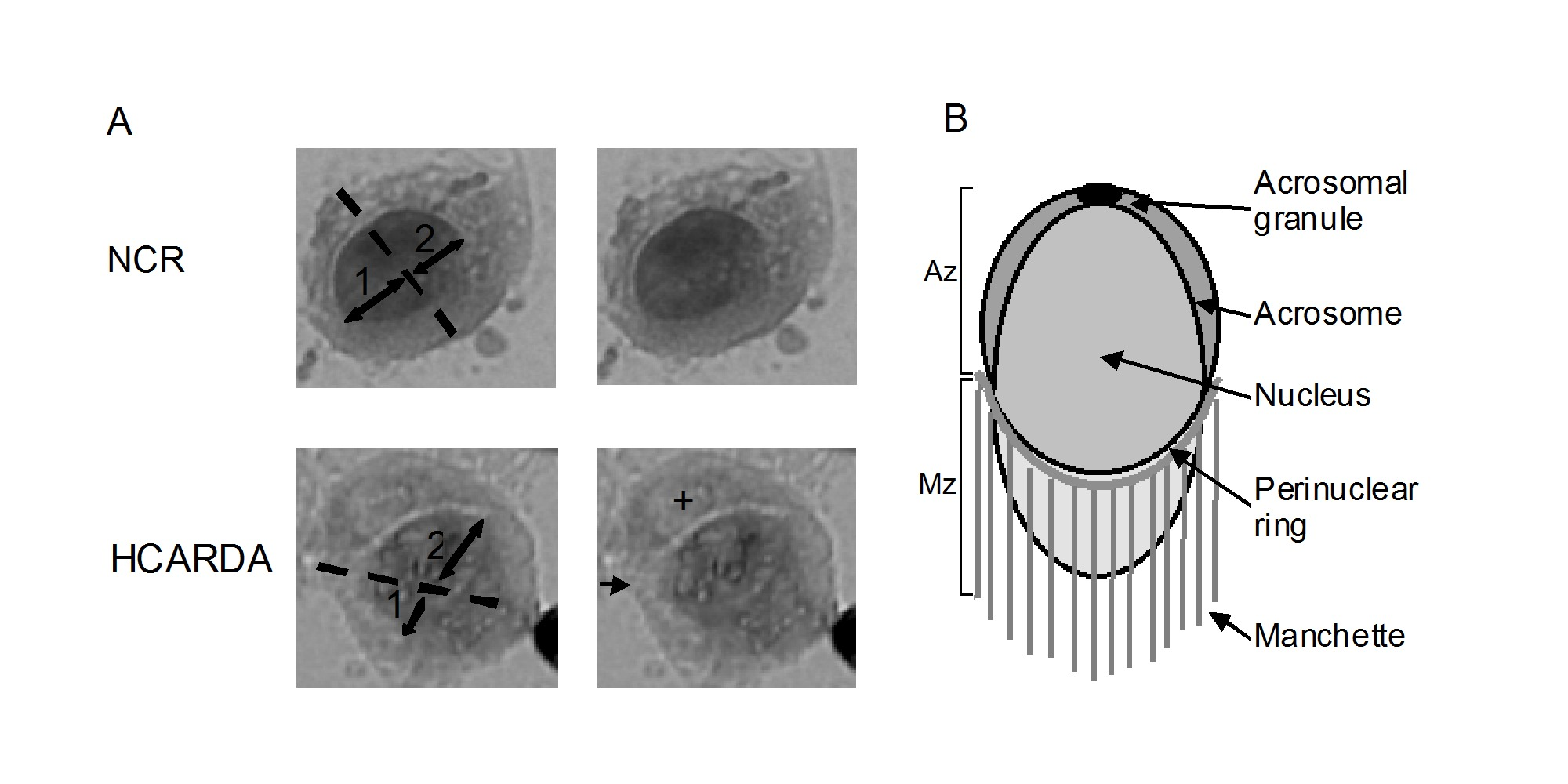

Supplement: S1 Fig — A: Spermatogenic cells isolated from seminiferous tubules. Dashed lines indicate the central axis and numbers 1 and 2 the distances from each acrosomal end (Left column). Note the presence of a vacuole in the spermatid´s cytoplasm of HCARDA (*), displacing the acrosomal granule (black arrow). 650X. B: Diagrammatic representation of an elongated spermatid. The microtubule-containing manchette (manchette zone = Mz) is inserted in a perinuclear ring to produce the stretching of acrosome around the nucleus (acrosomal zone = Az). (TIF) [file pone.0172994.s001.tif]

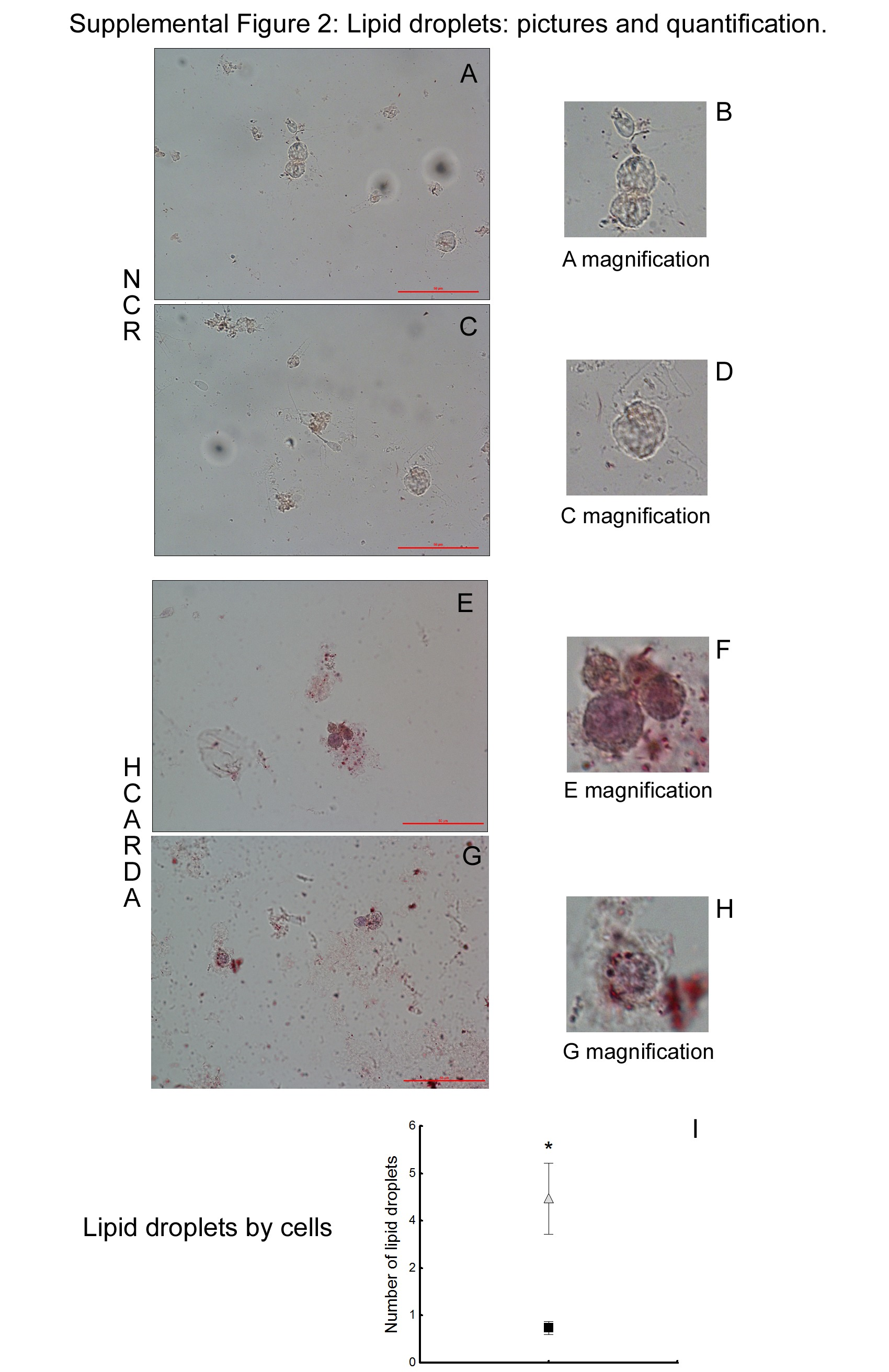

Supplement: S2 Fig — A—H: Isolated cells from seminiferous tubules showing neutral lipids stained with ORO. A–D: NCR; E–G: HCARDA. Bars represent 50 μm. Right column corresponds to magnification of some positive cells (B, D, F, G). I: Quantification of lipid droplets inside spermatogenic cells from NCR (■, 0.886 ± 0.331) and HCARDA (Δ, 4.158 ± 1.808) of 5 different experiments (Mean ± SD* = p ≤ 0. 01). (TIF) [file pone.0172994.s002.tif]
